# Discovery of a Novel Antifungal Agent in the Pathogen Box

**DOI:** 10.1128/mSphere.00120-17

**Published:** 2017-04-12

**Authors:** François L. Mayer, James W. Kronstad

**Affiliations:** Michael Smith Laboratories, Department of Microbiology and Immunology, University of British Columbia, Vancouver, British Columbia, Canada; Carnegie Mellon University

**Keywords:** antifungal drug, *Candida albicans*, *Cryptococcus gattii*, *Cryptococcus neoformans*, Pathogen Box

## Abstract

Human fungal pathogens cause over 2 million infections per year and are major drivers of morbidity and mortality. *Cryptococcus neoformans* and *Candida albicans* are two of the most common fungal pathogens of humans, together accounting for a staggering 1.4 million infections annually, with very high mortality rates. Patients with dysfunctional immune systems, such as individuals with HIV/AIDS, are particularly susceptible to fungal infections. Unfortunately, relatively few antifungal drugs are currently available and fungi frequently develop resistance, further complicating treatment approaches. In this study, we screened the Pathogen Box chemical library (Medicines for Malaria Venture, Switzerland) in an effort to identify novel antifungal compounds. This approach led to the discovery of a novel, highly potent antifungal agent with activity against both *C. neoformans* and *C. albicans*. Our initial study of the mechanism of action suggested that this novel compound prevents fungal proliferation by targeting the ability of *C. neoformans* to withstand stress at the plasma membrane and cell wall. Because this compound had previously been shown to have low toxicity for mammalian cells, we propose that it represents an attractive lead compound for further antifungal drug development.

**IMPORTANCE** *Cryptococcus neoformans* and *Candida albicans* are two major human fungal pathogens and together account for over 1.4 million infections annually, with very high mortality rates. These fungi often infect immunocompromised individuals, such as HIV/AIDS patients. In an effort to identify novel drugs with antifungal activity, we have screened the Pathogen Box for compounds with anticryptococcal and anticandidal activities. This approach led to the discovery of a promising lead compound (MMV688271) with strong antifungal potency under nutrient-limited conditions.

## Abstract (precis)

*Cryptococcus neoformans* and *Candida albicans* are two major human fungal pathogens and together account for over 1.4 million infections annually, with very high mortality rates. These fungi often infect immunocompromised individuals, such as HIV/AIDS patients. In an effort to identify novel drugs with antifungal activity, we have screened the Pathogen Box for compounds with anticryptococcal and anticandidal activities. This approach led to the discovery of a promising lead compound (MMV688271) with strong antifungal potency under nutrient-limited conditions.

## INTRODUCTION

Fungal pathogens have recently been estimated to contribute to as many human deaths as malaria or tuberculosis (1, 2). *Cryptococcus neoformans* and *Candida albicans* are two of the major human fungal pathogens, together accounting for over 1.4 million infections annually. Mortality rates vary between 20 and 75% but are generally very high (2). *C. neoformans* alone, for example, is estimated to cause over 600,000 deaths per year (3). Usually, a weakened immune system predisposes people to fungal diseases. Indeed, HIV/AIDS patients, individuals with severe burns, patients undergoing chemotherapy, and neonates are particularly at risk of such infections (2).

*C. neoformans* is naturally found in the environment on trees, in soil, and in bird droppings (4). In general, the fungus establishes an initial pulmonary infection upon inhalation of spores or desiccated yeast cells, with subsequent dissemination to the brain to cause meningoencephalitis (5). *C. neoformans* usually does not infect immunocompetent individuals; however, a related species, *Cryptococcus gattii*, recently caused an outbreak of disease in both immunocompromised and immunocompetent people (6).

*C. albicans* is not found in the environment but is obligately associated with warm-blooded animals. In humans, *C. albicans* is usually a harmless commensal and part of the natural microbiota. Upon immune suppression in humans, however, this fungus can switch to become an aggressive pathogen that causes a range of diseases including superficial mucosal and deep-seated systemic infections (7–9).

Currently, only a very small number of antifungal drugs are available for treatment of fungal diseases (10). Moreover, very few new compounds are in preclinical development or undergoing clinical trials (11). In the face of rapidly evolving resistance, it is therefore very important to identify novel antifungal drugs. One major obstacle in antimicrobial drug development is the necessity for specific activity toward pathogen-unique targets. For example, the echinocandins were the last new class of antifungal drugs introduced into the market, and these agents target the fungal cell wall, a structure not found in human cells. This specificity therefore reduces the potential for toxic side effects. In general, biologically relevant antimicrobials are either natural products produced by certain microorganisms or they are chemically synthesized (12).

The Pathogen Box is a project led by Medicines for Malaria Venture (MMV, Switzerland; http://www.pathogenbox.org/) that aims to identify novel drugs with activity against diseases such as tuberculosis, malaria, toxoplasmosis, and dengue, among others. The box consists of 400 mostly novel synthetic chemicals that were initially selected from a set of ~4 million compounds due to their low toxicity for mammalian cells and activity against specific microbial pathogens. In fact, the compounds display cytotoxicity at levels that are thought to be reasonable for drug discovery programs (http://www.pathogenbox.org/).

In this study, we screened the Pathogen Box compounds for antifungal activity against *C. neoformans* and *C. albicans*. This screen led to the discovery of a novel agent that had fungicidal potency under nutrient-limited conditions. Detailed time course, microscopic, and genetic analyses then suggested that this compound likely inhibits fungal growth via targeting the fungal response to stress at the plasma membrane and cell wall. We propose that this novel compound represents a promising candidate for further antifungal drug development.

## RESULTS

### A screen of the Pathogen Box identified compounds with antifungal activity.

**FIG 1:**
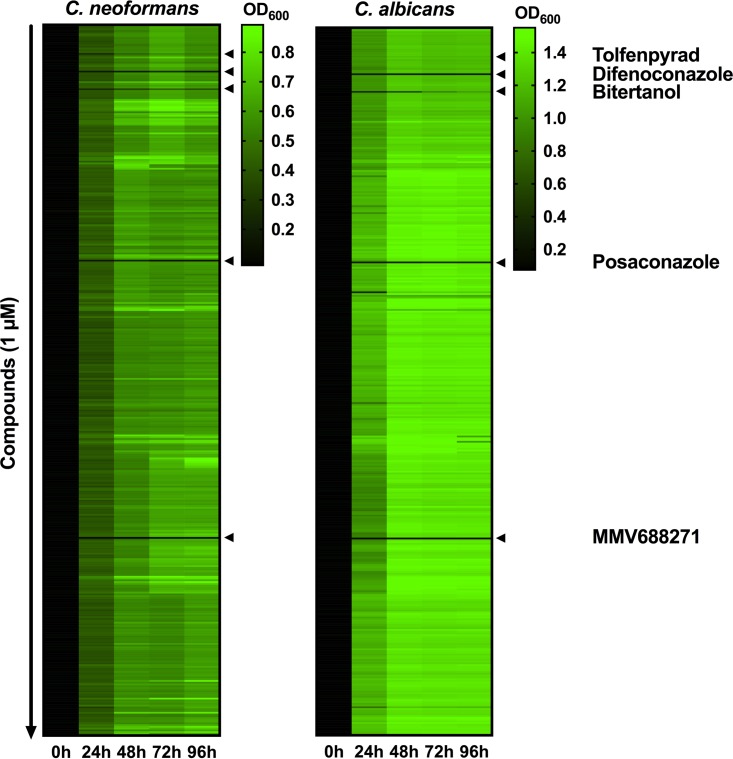
Screening of the Pathogen Box identified compounds with activity against human fungal pathogens. Each of the 400 compounds of the Pathogen Box (MMV, Switzerland) was analyzed for its impact on growth of *C. neoformans* wild-type strain H99 and *C. albicans* wild-type strain SC5314. Cultures were grown in YNB medium at 30°C with shaking for the indicated time periods, and growth was evaluated via measurements of the OD_600_. Results are represented as a heat map, with green indicating growth and black indicating no growth. All compounds were analyzed at a final concentration of 1 µM. The first and second rows from the tops of both heat maps represent results for the no-compound control and vehicle control, respectively. Black triangles at the sides of the heat maps indicate compounds with antifungal activity. Note that the novel compound MMV688271 completely prevented fungal proliferation.

We screened the 400 Pathogen Box compounds for their influence on fungal growth in yeast nitrogen base (YNB) medium, a defined minimal medium. Our rationale was that, with the exception of the gastrointestinal tract, it is assumed that most human body niches represent rather nutrient-limited environments for microbes. Indeed, a key defense strategy against pathogens, termed nutritional immunity, relies on keeping the amounts of available micronutrients (especially iron) at low levels to restrict microbial proliferation. Our screen at a relatively low drug concentration of 1 µM identified five compounds (tolfenpyrad, difenoconazole, bitertanol, posaconazole, and MMV688271) that displayed antifungal activity against the wild-type H99 strain of *C. neoformans* and the wild-type SC5314 strain of* C. albicans* (Fig. 1; Table 1). Interestingly, four of these five compounds had initially been shown to possess antikinetoplastid activity (http://www.pathogenbox.org/) (see Fig. S1A and B in the supplemental material). Tolfenpyrad is an insecticide used in agriculture and has known antifungal side effect activities. In our screen, it modestly inhibited the growth of both pathogens. Difenoconazole and bitertanol are used as fungicides in agriculture, and both target synthesis of the fungal membrane sterol, ergosterol. Difenoconazole completely prevented fungal proliferation, while bitertanol delayed but did not prevent fungal growth over the investigated time frame. Posaconazole is an important antifungal drug used in the clinic to treat fungal diseases, including infections with *C. albicans*. Similar to difenoconazole and bitertanol, this drug targets fungal ergosterol biosynthesis. As expected, posaconazole potently inhibited the growth of both fungi. Finally, the novel compound MMV688271 showed strong potency in preventing the growth of both *C. neoformans* and *C. albicans*. Similar to treatments with posaconazole, MMV688271 completely inhibited fungal proliferation (Fig. 1). Due to this finding and the novelty of the compound, we chose to focus on MMV688271 for further characterization.

10.1128/mSphere.00120-17.1 FIG S1  Chemicals with antifungal activity mainly fall into the category of kinetoplastid-targeting compounds. (A) Pathogen Box compounds grouped into different categories according to the activities displayed against initial pathogenic organisms (https://www.pathogenbox.org/). Compounds with antifungal activity identified in this study are marked in red. (B) Proportion of kinetoplastid-targeting compounds with antifungal activity. Download FIG S1, PDF file, 0.03 MB. Copyright © 2017 Mayer and Kronstad. 2017 Mayer and Kronstad This content is distributed under the terms of the Creative Commons Attribution 4.0 International license.

**TABLE 1:** Overview of Pathogen Box compounds with antifungal activity

Pathogen Box ID no.	Common name	Chemical formula	Commercial use	Antifungal target	Reference
MMV688934	Tolfenpyrad	C_21_H_22_N_3_O_2_Cl	Insecticide	Complex I of ETC^a^	Nihon Nohyaku Co., Ltd.
MMV688943	Difenoconazole	C_19_H_17_N_3_O_3_Cl_2_	Fungicide	Ergosterol synthesis	Syngenta (36)
MMV688942	Bitertanol	C_20_H_23_N_3_O_2_	Fungicide	Ergosterol synthesis	Bayer AG
MMV688774	Posaconazole	C_37_H_42_N_8_O_4_F_2_	Antifungal drug	Ergosterol synthesis	37
MMV688271		C_18_H_16_N_6_OCl_2_		Cell wall/membrane	This study

a ETC, electron transport chain.

### MMV688271 is a novel antifungal compound.

**FIG 2:**
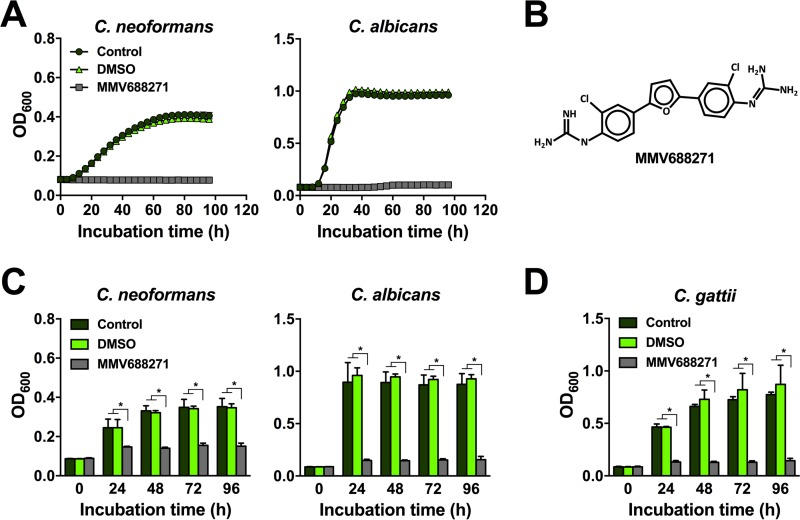
MMV688271 is a novel antifungal agent. (A) Growth curve analysis for *C. neoformans* H99 and *C. albicans* SC5314 in YNB medium at 30°C in the absence (control) or presence of 1 µM MMV688271, or the vehicle control (DMSO). MMV688271 blocked growth of both fungal pathogens. Results are means ± standard errors of the means of two independent experiments, each performed at least in duplicate. (B) Chemical structure of MMV688271. (C) MMV688271 inhibited fungal growth at 37°C. *C. neoformans* H99 or *C. albicans* SC5314 cells were incubated in YNB medium at 37°C in the absence (control) or presence of 1 µM MMV688271 or the vehicle control (DMSO). Results are means + standard deviations (SD) of two independent experiments, each performed in duplicate. *, *P* < 0.0001. (D) MMV688271 inhibits growth of the fungal pathogen *C. gattii*. The *C. gattii* wild-type strain R265 was grown in YNB medium at 30°C in the absence (control) or presence of 1 µM MMV688271 or the vehicle control (DMSO). Results are the means + SD of two independent experiments, each performed in duplicate. *, *P* < 0.0001.

We next performed automated growth analyses using a microtiter plate reader and confirmed that MMV688271 potently inhibited the growth of *C. neoformans* and *C. albicans* at 30°C in YNB medium (Fig. 2A). Interestingly, when we performed an analogous experiment using nutrient-rich YPD medium, MMV688271 had no detectable effect on fungal growth (Fig. S2). This indicated that the antifungal mechanism of MMV688271 is dependent on a nutrient-limited environment. As shown in Fig. 2B, MMV688271 is 1,1′-[2,5-furandiyl bis(2-chloro-4,1-phenylene)]diguanidine (ChemSpider molecule ID 23156441) with a predicted mass of 403.265 Da. To investigate whether the compound also exerts antifungal activity at a human physiological temperature, we performed a time course experiment at 37°C in YNB. At this elevated temperature, MMV688271 also efficiently blocked growth of both *C. neoformans* and *C. albicans* (Fig. 2C). These results support the idea that MMV688271 may be effective for human therapy. We also tested the influence of MMV688271 on *C. gattii*, a species related to *C. neoformans* that recently caused a significant outbreak of cryptoccocal infection on Vancouver Island and in the northwestern United States (6). Time course assays with MMV688271 revealed that this compound efficiently blocked the growth of the *C. gattii* outbreak strain R265, thereby broadening the spectrum of fungal pathogens that are potential targets (Fig. 2D).

10.1128/mSphere.00120-17.2 FIG S2  Analysis of the growth of *C. neoformans* H99 and *C. albicans* SC5314 in YPD medium at 30°C in the absence (control) or presence of 1 µM MMV688271 or the vehicle control (DMSO). In comparison to minimal YNB medium, the compound did not show antifungal activity in complex YPD medium. Results are means ± standard errors of the means of two independent experiments, each performed at least in duplicate. Download FIG S2, PDF file, 0.04 MB. Copyright © 2017 Mayer and Kronstad. 2017 Mayer and Kronstad This content is distributed under the terms of the Creative Commons Attribution 4.0 International license.

### MMV688271 has fungicidal activity.

**FIG 3:**
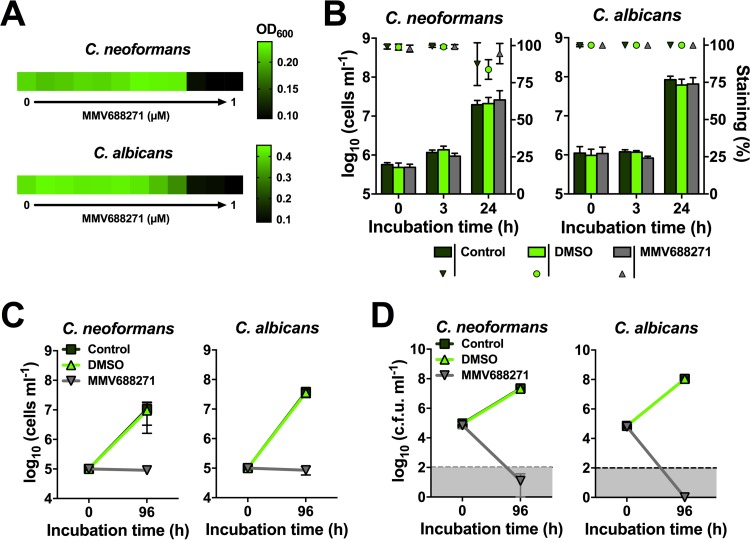
MMV688271 has fungicidal activity at lower cell concentrations. (A) The MIC_50_ (defined here as the concentration required to inhibit at least 50% of fungal growth) of MMV688271 against *C. neoformans* H99 or *C. albicans* SC5314 at 30°C is 250 nM. Fungal cells were exposed to different compound concentrations for 24 h, and growth was evaluated via measurements of the OD_600_. Results are presented as a heat map, with green indicating growth and black indicating no growth. Compound dilution steps were 2-fold, with 1 µM as the highest concentration. The experiment was performed twice in duplicate with similar results. The results from one experiment are shown. (B) With higher fungal inocula (10^6^ cells ml^−1^), exposure to MMV688271 did not affect fungal cell viability. *C. neoformans* H99 or *C. albicans* SC5314 cells were treated with 1 µM compound, and cell numbers (bars; left *y* axis) as well as methylene blue staining (an indicator of cell viability; values on the right *y* axis) were assessed. The staining of MMV668271-exposed cells was comparable to that of unexposed control cells. Note that fungal growth was only modestly inhibited by the compound in this experiment, as a 10-fold-higher initial inoculum was used to allow the analysis of cell numbers. The results are means + standard deviations (SD) of two independent experiments, each performed in duplicate. (C) Exposure of fungal cells to MMV688271 did not lead to cell lysis. *C. neoformans* H99 or *C. albicans* SC5314 cells were exposed to 1 µM MMV668271 for 96 h, and the final cell numbers were determined. Note that exposure to the compound prevented fungal growth but did not lead to a reduction of cell numbers from the original inoculum. Results are means ± SD of two independent experiments, each performed in duplicate. (D) A CFU-based analysis revealed that MMV688271 has fungicidal activity. *C. neoformans* H99 or *C. albicans* SC5314 cultures (10^5^ cells ml^−1^ each) were exposed to 1 µM MMV668271 for 0 h and 96 h, and CFU were determined from serial dilutions. Treatment with the compound resulted in an at least a 1,000-fold reduction in cell viability. Note that due to the relatively low culture volume (200 µl), the detection limit for this experiment was 10^2^ cells ml^−1^ (dotted line). Results are means ± SD of two independent experiments, each performed at least in duplicate.

To further characterize the antifungal activity of MMV688271, we next determined its MIC. A broth microdilution assay in YNB medium at 30°C revealed that the compound had a MIC_50_ value of 250 nM (~0.12 µg/ml) for both *C. neoformans* and *C. albicans* (Fig. 3A). When we performed the experiment at 37°C, we also obtained MIC_50_ values of 250 nM for both fungi (Fig. S3). These results again indicated that MMV688271 maintains potent antifungal activity even at an elevated temperature. We next used methylene blue staining to quantify viability following exposure to the compound. This dye preferentially stains dead cells. We used a 10-fold-higher initial inoculum (2 × 10^5^ cells per well of a 96-well plate) in this experiment to have enough cells for quantification. Exposure of *C. neoformans* and *C. albicans* to the compound and quantification of both cell numbers and dead cells after 3 h and 24 h revealed that MMV688271 did not influence cell viability as measured by staining (Fig. 3B). We also noted that the compound did not fully inhibit fungal growth at 1 µM in this experiment, indicating that the antifungal activity is not only medium dependent but also dependent on the level of the initial fungal inoculum. To further determine if MMV688271 is fungistatic or fungicidal, we next analyzed whether exposure to the compound resulted in cell lysis, as determined microscopically with a hemocytometer. Analysis of the numbers of cells in *C. neoformans* and *C. albicans* cultures exposed to the compound for 96 h revealed that, although MMV688271 prevented fungal proliferation, cell numbers at the end of the experiment were similar to those in the starting inoculum (Fig. 3C). In contrast, the number of control cells incubated under the same conditions increased by approximately 1,000-fold in the absence of MMV688271 (Fig. 3C). These results indicated that MMV688271 does not provoke cell lysis. To directly investigate if the compound has fungistatic or fungicidal activity, we also performed an assay based on the enumeration of CFU (Fig. 3D). This experiment revealed that MMV688271-treated fungal cells lost viability by a factor of at least 1,000 within 96 h of incubation. In contrast, control and dimethyl sulfoxide (DMSO)-exposed cells proliferated, and cell numbers rose from 10^5^ cells ml^−1^ to approximately 10^8^ cells ml^−1^ (Fig. 3D). Overall, these results indicated that MMV688271 has fungicidal activity with low MIC_50_s against *C. neoformans* and *C. albicans*.

10.1128/mSphere.00120-17.3 FIG S3  The MIC_50_ of MMV688271 against *C. neoformans* H99 or *C. albicans* SC5314 at 37°C is 250 nM. Fungal cells were exposed to different drug concentrations for 24 h, and growth was evaluated via OD_600_ measurements. Results are presented as a heat map, with green indicating growth and black indicating no growth. Drug dilution steps were 2-fold, with 1 µM as the highest concentration. The experiment was performed twice in duplicate with similar results. Results from one experiment are shown. Download FIG S3, PDF file, 0.03 MB. Copyright © 2017 Mayer and Kronstad. 2017 Mayer and Kronstad This content is distributed under the terms of the Creative Commons Attribution 4.0 International license.

### MMV688271 may target the response to stress at the plasma membrane and cell wall.

**FIG 4:**
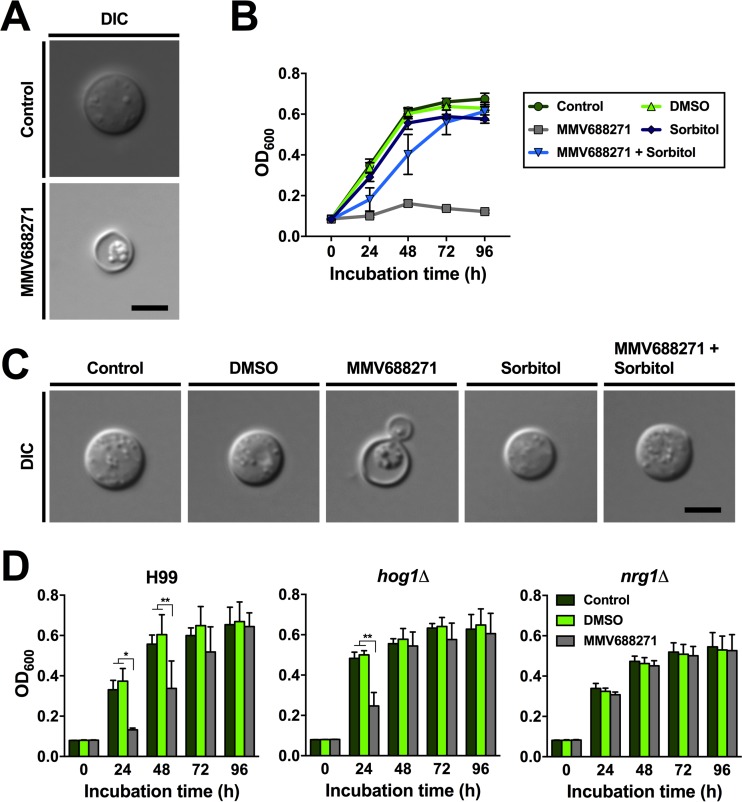
MMV688271 may target fungal cells via increased susceptibility to cell wall and/or membrane stress. (A) Microscopic analysis of *C. neoformans* H99 cells following exposure to the compound for 96 h in YNB medium. Compared to the control, MMV688271-exposed cells displayed aberrant cell morphologies with shrunken cytoplasm. Bar, 2 µm. (B) The cell membrane-stabilizing agent sorbitol bypassed MMV668271-dependent growth inhibition. The growth of *C. neoformans* H99 at 30°C in YNB medium with or without the test compound and/or sorbitol was analyzed. Results are the means ± standard errors of the means of two independent experiments, each performed in duplicate. (C) DIC microscopy of cells from panel B after 96 h of incubation, showing that sorbitol rescues normal cell morphology of compound-exposed cells. Bar, 2 µm (all micrographs). (D) The mutants *hog1*Δ and *nrg1*Δ had impaired responses to stresses and changes in cell wall integrity, respectively, and displayed reduced susceptibility profiles upon exposure to MMV668271 compared to the wild-type strain H99. Fungal cells were exposed to 500 nM MMV688271 in YNB medium, and growth was assessed by OD_600_ measurements. Note that the *hog1*Δ mutant adapted more rapidly to the compound than the wild-type strain (48-h time point) and that the *nrg1*Δ mutant was completely tolerant to MMV668271 at this concentration. Results are means + standard deviations of two independent experiments, each performed in duplicate. *, *P* < 0.01; **, *P* < 0.001.

We next initiated an investigation of the potential mechanism of action for MMV688271 with a focus on *C. neoformans*. Microscopic analysis revealed that cells exposed to the compound had an aberrant appearance that included a dense, shrunken cytoplasm with tightly packed organelles compared to control cells (Fig. 4A). This phenotype is reminiscent of cells undergoing apoptosis, and this was consistent with the observed loss of viability upon MMV668271 treatment, as mentioned above (Fig. 3) (13, 14). Apoptosis is one aspect of the response to stress and can be associated with oxidative stress (15). It has also been proposed that certain antibiotics can induce oxidative stress in bacterial and fungal cells (16–18). To address the possibility that MMV68271 induces oxidative stress, we hypothesized that mutants with defects in catalase (*cat1*Δ) or superoxide dismutase (*sod1*Δ) activity might display enhanced sensitivity toward the compound compared to the wild-type strain. Though not statistically significant, both a *cat1*Δ and a *sod1*Δ mutant displayed a trend toward increased susceptibility to MMV688271 compared to the wild type (Fig. S4). As the response to stress is known to be regulated by the cyclic AMP/protein kinase A (cAMP/PKA) pathway in fungi (19), we also investigated the growth dynamics of a mutant with a deletion in the gene encoding the cAMP-dependent protein kinase catalytic subunit Pka1 (*pka1*Δ). However, no significant differences were observed between the growth of this mutant and the wild type upon exposure to MMV688271 (Fig. S4).

10.1128/mSphere.00120-17.4 FIG S4  Disruption of *C. neoformans* genes for catalase (*CAT1*) or superoxide dismutase (*SOD1*) resulted in a non-statistically significant trend toward increased MMV688271 susceptibility compared to the wild-type strain (see 48-h and 72-h time points). Deletion of the gene encoding the cAMP-dependent protein kinase catalytic subunit (PKA1) did not impact growth in the presence of MMV688271. The wild-type strain H99 (A) and the *cat1Δ* (B), *sod1Δ* (C), and *pka1Δ* (D) mutants were exposed to 500 nM MMV688271, and growth was assessed based on OD_600_ measurements (E). Data comparison from panel A is for MMV688271-exposed strains only. Results are the means + standard deviations of at least two independent experiments, each performed in duplicate. *, *P* < 0.05; **, *P* < 0.01; ***, *P* < 0.001; ns, not significant. Download FIG S4, PDF file, 0.04 MB. Copyright © 2017 Mayer and Kronstad. 2017 Mayer and Kronstad This content is distributed under the terms of the Creative Commons Attribution 4.0 International license.

Several known antifungal drugs, such as echinocandins or azoles, target the cell wall or plasma membrane, respectively, and these agents induce a variety of stresses, including the formation of reactive oxygen species (ROS) (20). We hypothesized that MMV688271 may also target the cell wall or plasma membrane to influence the ability of cells to withstand stress. To test this idea, we employed sorbitol (250 mM) as an osmotic stabilizer to potentially rescue cells experiencing membrane and cell wall stress as a result of MMV688271-mediated growth inhibition (21, 22). Indeed, when we coincubated cells in YNB medium containing both MMV668271 and sorbitol, fungal growth was restored to levels similar to those seen in unexposed cells. Treatment of cells with sorbitol alone had no impact on growth (Fig. 4B). A microscopic analysis confirmed these findings, as it revealed that cells coexposed to MMV668271 and sorbitol had a wild-type appearance (Fig. 4C). These results strongly suggested that a stress response involving the cell surface may indeed be targeted by MMV688271. To further investigate this possibility, we used *hog1*Δ and *nrg1*Δ mutants, which influence the response to a variety of stresses and cell wall integrity, and performed time course analyses with 500 nM MMV688271 in YNB medium. We used this reduced concentration of the test compound in order to have a better resolution of hypo- or hypersusceptibility. The *hog1*Δ mutant showed a trend toward reduced drug susceptibility at 24 h compared to the wild-type control and had significantly reduced susceptibility at 48 h (Fig. 4D). Surprisingly, the *nrg1*Δ mutant was completely tolerant to MMV688271 at each of the time points investigated (Fig. 4D). An analogous experiment performed in nutrient-rich YPD medium at an MMV668271 concentration of 1 µM did not result in observable activity for either strain (Fig. S5). Together, these results indicate that at least part of the fungicidal activity of MMV668271 is due to changes in cell wall or membrane functions that increase the susceptibility of treated cells to stress under nutrient-limited conditions.

10.1128/mSphere.00120-17.5 FIG S5  The *C. neoformans* wild-type strain H99 and the mutants *hog1Δ* and *nrg1Δ* displayed similar growth kinetics in the presence of MMV688271 in YPD medium. Fungal cells were exposed to 500 nM MMV688271, and growth was assessed based on OD_600_ measurements. Results are means + standard deviations of two independent experiments, each performed in duplicate. *, *P* < 0.05. Download FIG S5, PDF file, 0.03 MB. Copyright © 2017 Mayer and Kronstad. 2017 Mayer and Kronstad This content is distributed under the terms of the Creative Commons Attribution 4.0 International license.

## DISCUSSION

The Pathogen Box is the follow-up project to the Malaria Box, which comprised 400 compounds selected from a screen of over 6 million chemicals (23). Both projects are based on an open source drug discovery approach and involve testing the compounds against specific human pathogens by a large number of laboratories around the world. For the Malaria Box, this approach and the combined efforts of over 100 laboratories led to the identification of a potential mechanism of action for over 130 drugs with antimalaria activity (24). Importantly, this screen also led to the discovery of compounds with antimicrobial potency against pathogens other than the malaria parasite.

Pathogenic microbes usually infect or transition through human body niches that are poor in nutrients. Indeed, sequestering nutrients such as iron is a host strategy to control microbial proliferation, and this process has been termed nutritional immunity (25). Both *C. neoformans* and *C. albicans* are exposed to nutrient-limited environments during infection, for example, during interactions with host epithelial and endothelial cells or with immune cells such as macrophages and neutrophils (26, 27). We therefore screened the Pathogen Box compounds in YNB, a minimal, nutrient-limited medium. Surprisingly, we found that MMV688271 had a strong capacity to inhibit fungal growth in this medium, but not in nutrient-rich YPD medium (Fig. 2A; see also Fig. S2 in the supplemental material). We performed the screening and all further experiments using a relatively low drug concentration of 1 µM. We hypothesized that this low concentration would lead to identification of the most potent compounds with antifungal activity. As we identified the cell wall and plasma membrane as possible targets of MMV688271 action, one explanation for the observed differences in activity in YNB versus YPD medium could be alterations in fungal cell wall architecture in response to growth in these different media. Such media effects on the cell wall have been described for *C. albicans*, for example, when cells are grown in glucose versus lactate as a carbon source (28).

The Pathogen Box contains two reference antifungal drugs, amphotericin B and posaconazole. We found the latter drug to have strong antifungal activity in our initial screen, but we did not identify amphotericin B, probably due to the low drug concentration used. Our library screen also identified one insecticide with known antifungal side effects (tolfenpyrad) and two fungicides (bitertanol and difenoconazole), which validated our screening approach (Fig. 1). However, we did not further investigate these three compounds, because they are already used in agriculture, and microbes (especially environmental microbes such as *C. neoformans* and *C. gattii*) may be more likely to have developed resistance due to prior exposure.

We identified strong antifungal activity for MMV688271, and previous experiments conducted by MMV and the Laboratory of Microbiology, Parasitology and Hygiene (LMPH) at the University of Antwerp also detected antimicrobial activities against the human parasites *Trypanosoma cruzi*, *Trypanosoma brucei*, and *Leishmania infantum*. To the best of our knowledge, however, no antifungal activity has been described for this compound so far. We note with interest that four of the five identified compounds from our screen fall into the category of kinetoplastid-targeted drugs (Fig. S1) (http://www.pathogenbox.org/). This observation suggests that fungi and kinetoplastids may share common antimicrobial drug targets and that potential synergy between the fungal and parasite research fields may propel drug discovery.

In toxicity analyses conducted by MMV, LMPH, and AbbVie, MMV688271 had been shown to have CC_50_s (50% cytotoxicity concentrations) of 15.4 µM against the lung tissue cell line MRC-5, 16 µM against peritoneal murine macrophages (PMM), and 13.5 µM against the liver hepatocellular carcinoma-derived HepG2 cell line (http://www.pathogenbox.org/). We determined an MIC_50_ of 250 nM for both *C. neoformans* and *C. albicans* (Fig. 3A). Therefore, MMV688271 displays antifungal activity well below concentrations that are toxic to human cells. Moreover, this compound also showed potent antifungal activity at the physiological body temperature of 37°C (Fig. 2C; see also Fig. S3 in the supplemental material).

We found that MMV688271 not only inhibited the growth of *C. neoformans* and *C. albicans*, but also that of *C. gattii* (Fig. 2D). This finding suggests that this agent has broad-spectrum anticryptococcal activity and hints at a conserved antifungal target. We also determined that MMV688271 has fungicidal activity at lower fungal cell densities (Fig. 3D). At higher cell concentrations, however, the compound appeared to mainly exert fungistatic activity, as determined by methylene blue staining (Fig. 3B). Therefore, this agent may not be suitable to completely eradicate fungal infections depending on pathogen burden. However, in the case of *C. albicans*, which is part of the normal human microbiota, inhibiting fungal growth, rather than complete sterilization, may be more desirable under certain circumstances. Also, MMV688271 may be used in combination therapies to treat infections caused by other fungi, such as *C. neoformans*, or the compound may be used prophylactically.

We have identified the plasma membrane and the cell wall as possible targets of MMV688271, based on microscopic, phenotypic, and genetic analyses (Fig. 4). Cells exposed to MMV688271 had an aberrant appearance that included a dense, shrunken cytoplasm, indicative of oxidative and osmotic stress as well as apoptosis (13–15). We did observe a trend toward increased susceptibility to MMV688271 for mutants with defects in ROS-detoxifying enzymes (Fig. S4). As ROS can be induced via cell wall- and cell membrane-targeted stresses, we also hypothesized that MMV688271 may also target these structures. Two key pieces of evidence support an impact of MMV688271 on the fungal response to stress at the cell surface. First, osmotic stabilization by sorbitol rescued growth inhibition by the compound and, second, a defect in the transcription factor Nrg1 blocked susceptibility. Nrg1 is known to regulate genes that encode cell wall functions (e.g., chitin synthases) in *C. neoformans*, and an *nrg1* deletion mutant had elevated susceptibility to agents that challenge membrane and cell wall integrity (i.e., high concentrations of salt, sorbitol, and detergent) (29). A minor influence of deletion of the *HOG1* gene, which encodes a key stress response factor, was also noted. Interestingly, loss of Hog1 in *C. neoformans* is known to increase membrane ergosterol levels as well as to impact the response to a number of stresses (e.g., oxidative and temperature stresses, heat shock, etc.) (30). In that context, it is possible that loss of Nrg1 or Hog1 provokes compensatory adaptations that reduce susceptibility to MMV688271. Interestingly, membrane effects appear to be a theme in our screening, because three of the inhibitory compounds that we identified influence ergosterol synthesis (difenoconazole, bitertanol, and posaconazole).

In general, the fungal cell wall is a key target in current antifungal drug development efforts (11). For example, the echinocandins, such as caspofungin or anidulafungin, inhibit biosynthesis of (1,3)-β-d-glucan, an essential component of the fungal cell wall (31). Interestingly, while it is potent against *C. albicans*, caspofungin is ineffective against *C. neoformans* (32). Therefore, MMV688271 may be a useful alternative to target functions related to the plasma membrane and cell wall in *C. neoformans*. However, further investigation will be required to identify the underlying mechanisms by which MMV668271 influences stress at the plasma membrane and cell wall.

Recently, the Pathogen Box compounds have been screened against *C. albicans* for antibiofilm activity (33). The authors of that study identified six molecules with biofilm-inhibitory activity, none of which was identified in our current study. This indicates that fungal cells grown as part of a biofilm or under planktonic conditions possess different susceptibilities toward antifungal drugs. Indeed, one of the six hit compounds that Vila and Lopez-Ribot identified showed increased activity against biofilm cells compared to planktonic cells (33). Moreover, those authors used a higher drug concentration (5 µM) for their initial screen than we used in our study, and the biofilm experiments were performed in RPMI 1640 medium (33). These differences may also have contributed to the fact that different anti-*C. albicans* compounds were identified in both studies.

In summary, we identified the compound MMV688271 as a novel antifungal agent with potential fungicidal activity under conditions of nutrient limitation. Due to its low MIC_50_ against major pathogenic fungi such as *C. neoformans* and *C. albicans* and its low toxicity against human cells, we propose that this compound may represent an attractive candidate for further drug development.

## MATERIALS AND METHODS

### Fungal strains and growth conditions.

*C. neoformans* var.* grubii* strain H99 (serotype A), *C. gattii* strain R265 (molecular type VGIIa, serotype B), and *C. albicans* strain SC5314 were used as wild-type controls. Other strains used in this study are listed in Table S1 in the supplemental material. Fungal strains were routinely maintained on YPD agar (1% yeast extract, 2% Bacto peptone, 2% d-glucose, 2% agar). Overnight cultures were grown in liquid YPD medium in a shaking incubator at 30°C and 180 rpm. Antifungal assays were performed in YNB minimal medium (2% dextrose, 0.17% yeast nitrogen base, 0.5% ammonium sulfate) or YPD, as indicated.

10.1128/mSphere.00120-17.6 TABLE S1  Fungal strains used in this study. Download TABLE S1, PDF file, 0.1 MB. Copyright © 2017 Mayer and Kronstad. 2017 Mayer and Kronstad This content is distributed under the terms of the Creative Commons Attribution 4.0 International license.

### The Pathogen Box compound library.

The Pathogen Box was kindly provided by Medicines for Malaria Venture (MMV, Switzerland) and contains 400 mostly novel drug-like compounds. Each compound was selected based on antimicrobial activity against a defined human pathogen (Fig. S1). Compounds were supplied in 96-well microtiter plates containing 10 µl/well of 10 mM compound dissolved in DMSO. The original samples were aliquoted to 10 individual library sets with a final drug concentration of 1 mM in DMSO, as recommended by MMV. All plates were stored at −80°C.

### Library screening for identification of compounds with antifungal activity.

Screening of the 400 compounds was conducted in sterile, polystyrene, flat-bottom, 96-well microtiter plates (Greiner). Overnight fungal cultures were washed twice in sterile phosphate-buffered saline (PBS), and 2 × 10^4^ cells were exposed to 1 µM drug in a final volume of 200 µl YNB or YPD per well. Cells in medium only and cells in medium plus vehicle only (final DMSO concentration, 0.1%) were included as controls. Plates were sealed with sterile adhesive foil to prevent evaporation and incubated under continuous shaking (200 rpm) at 30°C or 37°C. At the indicated time points, measurements of the optical density at 600 nm (OD_600_) were performed using a microplate reader (Infinite M200; Tecan). The resulting growth data were visualized quantitatively with color using Prism version 7.0 (GraphPad Software, Inc., USA).

### Growth curves.

A microplate reader (Infinite M200; Tecan) was used for automated growth curve analyses. Overnight fungal cultures were washed twice in sterile PBS, and 2 × 10^4^ cells were exposed to 1 µM MMV688271 in a final volume of 200 µl YNB or YPD in 96-well microtiter plates. Appropriate controls without compound and with vehicle (DMSO) were included, and plates were sealed with sterile adhesive foil. Growth of the strains was then recorded by measuring the OD_600_ at 30-min intervals for up to 96 h.

### Antifungal time course analysis.

Time course growth analyses were performed for confirmation of MMV688271 antifungal activity, for sorbitol rescue experiments and for *C. neoformans* mutant analysis. Fungal cultures were grown overnight in YPD and washed twice in PBS. Cell densities were adjusted to 2 × 10^4^ cells per 200 µl medium. Experiments were conducted in 96-well microtiter plates sealed with adhesive foil. Plates were incubated under continuous shaking (200 rpm) at 30°C or 37°C. A concentration of 250 mM sorbitol was used for sorbitol rescue experiments. The OD_600_ was determined with a microplate reader (Infinite M200; Tecan) at the indicated time points.

### Assessment of MICs.

Assays for the MIC of MMV688271 were performed in sterile 96-well microtiter plates (Greiner) by using a previously published broth microdilution approach (34, 35). Briefly, overnight fungal cultures were washed twice in sterile PBS and adjusted to 10^4^ cells ml^−1^ in YNB medium. In parallel, 96-well plates were prepared with a 2-fold dilution gradient of antifungal drug in YNB. Fungal cells were then added to the wells such that 10^3^ cells per well were exposed to the different drug concentrations in a total 200-µl volume. The drug concentrations used were 1 µM, 500 nM, 250 nM, 125 nM, 62.5 nM, 31.25 nM, 15.63 nM, 7.81 nM, 3.91 nM, 1.95 nM, and 0.98 nM. Cells exposed to medium only were included as controls. Plates were sealed with sterile adhesive foil and incubated under continuous shaking at 30°C or 37°C for 24 h. The OD_600_ was then measured using a microplate reader (Infinite M200; Tecan). The data were visualized quantitatively with color using Prism version 7.0 (GraphPad Software, USA).

### Analysis of cell lysis and cell viability.

For analysis of possible cell lysis following exposure to compounds, fungal cell numbers were determined microscopically at the end of the experiment (after 4 days) by using a hemocytometer (Neubauer). Cell numbers were compared to the initial inoculum of 2 × 10^4^ cells. Methylene blue staining was used for assessing fungal cell viability following exposure to MMV688271. Overnight fungal cultures were washed twice in sterile PBS and adjusted to 10^6^ cells ml^−1^ in YNB medium. In a sterile 96-cell microtiter plate, 2 × 10^5^ fungal cells were then exposed to 1 µM MMV688271 in a total volume of 200 µl. Plates were sealed with adhesive foil and incubated in a shaking incubator (200 rpm) at 30°C. After 0 h, 3 h, and 24 h, 10 µl of cells was mixed with 10 µl of sterile 0.05% methylene blue (Sigma) and incubated for 10 min at room temperature. Cell numbers and the proportion of dead/viable cells were then determined microscopically using a hemocytometer (Neubauer).

For analysis of cell viability by counting CFU after exposure to MMV688271, overnight fungal cultures were washed twice in sterile PBS and adjusted to 10^5^ cells ml^−1^ in YNB medium. In a sterile 96-well microtiter plate, 2 × 10^4^ fungal cells were then exposed to 1 µM MMV688271 in a total volume of 200 µl. Plates were sealed with adhesive foil and incubated under continuous shaking (200 rpm) at 30°C. At 0 h and 96 h, cultures were mixed by pipetting up and down, serially diluted, and plated onto YPD agar plates. Plates were then incubated at 30°C for 1 to 2 days, and CFU were determined. Note that due to the relatively low culture volume, cells could not be centrifuged and washed. To circumvent possible compound carryover effects on the CFU analyses, MMV688271-treated fungal cells were diluted at least 10-fold in sterile PBS before plating onto YPD medium. At this diluted concentration, the compound does not possess inhibitory activity (Fig. 3A) and therefore is not expected to affect colony formation.

### Microscopy.

Differential interference contrast (DIC) microscopy was performed with an Axioplan 2 imaging microscope (Zeiss); micrographs were captured with a Cool Snap HQ camera (Photometrics) and analyzed using MetaMorph software (Molecular Devices).

### Statistical analysis.

Data were visualized and statistically analyzed using Prism version 7.0 (GraphPad Software, USA). Statistical tests were performed by two-way analysis of variance followed by a Bonferroni correction. *P* values of ≤0.05 were considered significant.
