# Supplementary figures and images for: Discovery of a Novel Antifungal Agent in the Pathogen Box
Source: mSphere. 2017 Apr 12;2(2):e00120-17. doi: 10.1128/mSphere.00120-17 (PMC5390095; doi:10.1128/mSphere.00120-17)

**A**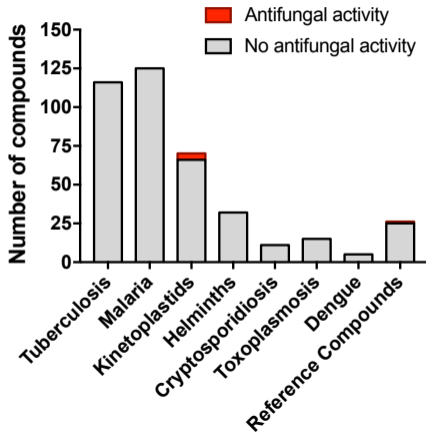**B**

### Kinetoplastid-targeted compounds

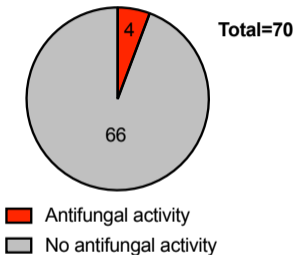

Supplement: FIG S1 [file sph002172266sf1.pdf]

*C. neoformans*

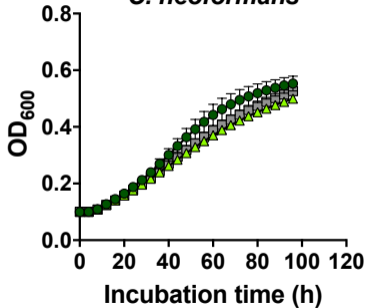

*C. albicans*

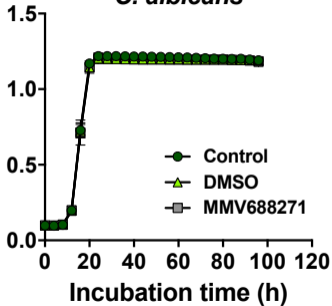

Supplement: FIG S2 [file sph002172266sf2.pdf]

# *C. neoformans*

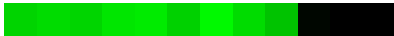

MMV688271 ( $\mu\text{M}$ )

OD<sub>600</sub>

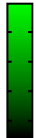

0.20  
0.18  
0.16  
0.14

# *C. albicans*

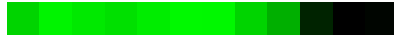

MMV688271 ( $\mu\text{M}$ )

OD<sub>600</sub>

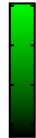

0.8  
0.6  
0.4  
0.2

Supplement: FIG S3 [file sph002172266sf3.pdf]

**A****H99**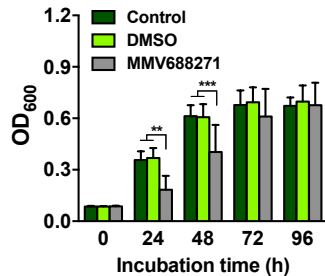**B*****cat1*Δ**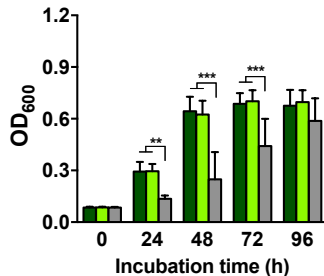**C*****sod1*Δ**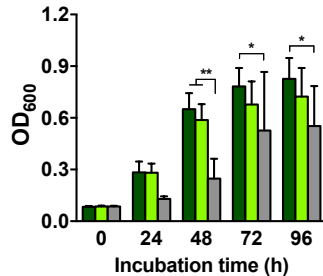**D*****pka1*Δ**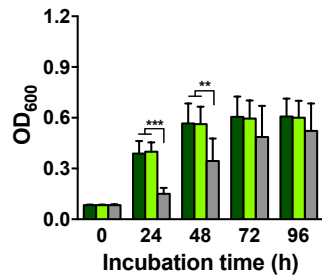**E****MMV688271**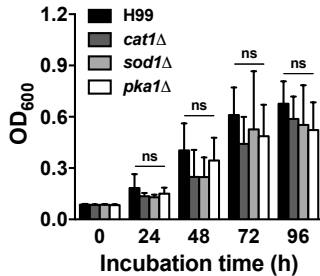

Supplement: FIG S4 [file sph002172266sf4.pdf]

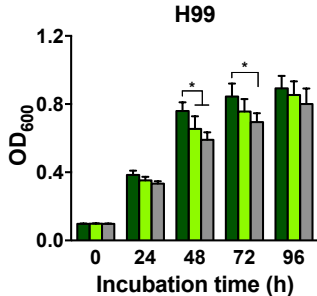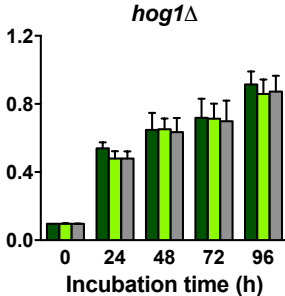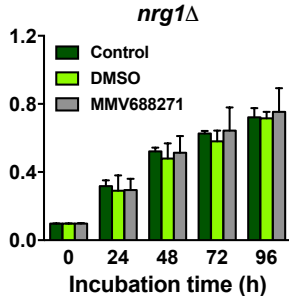

Supplement: FIG S5 [file sph002172266sf5.pdf]
