# Supplementary material for: Discovery of a Novel Antifungal Agent in the Pathogen Box
Source: mSphere. 2017 Apr 12;2(2):e00120-17. doi: 10.1128/mSphere.00120-17 (PMC5390095; doi:10.1128/mSphere.00120-17)
Supplement: TABLE S1 [file sph002172266st6.pdf]

Table S1 Fungal strains used in this study.

Organism	Strain	Genotype	Source, Reference(s)
C. neoformans	H99	Serotype A, *MAT*α	Joseph Heitman, USA (1)
	*hog1*Δ	*hog1*::*nat*	Hiten Madhani, USA
	*nrg1*Δ	*nrg1*::*neo*	Andrew Alspaugh, USA (2)
	*cat1*Δ	*cat1*::*ura5*	John Perfect, USA (3)
	*sod1*Δ	*sod1*::*ura5*	John Perfect, USA (4)
	*pka1*Δ	*pka1*::*ade2*	Joseph Heitman, USA (5)
C. gattii	R265	VGIIa	Karen Bartlett, Canada (6, 7)
C. albicans	SC5314	wild-type	Malcolm Whiteway, Canada (8)

References

1. Janbon G, Ormerod KL, Paulet D, Byrnes EJ, 3rd, Yadav V, Chatterjee G, Mullapudi N, Hon CC, Billmyre RB, Brunel F, Bahn YS, Chen W, Chen Y, Chow EW, Coppee JY, Floyd-Averette A, Gaillardin C, Gerik KJ, Goldberg J, Gonzalez-Hilarion S, Gujja S, Hamlin JL, Hsueh YP, Ianiri G, Jones S, Kodira CD, Kozubowski L, Lam W, Marra M, Mesner LD, Mieczkowski PA, Moyrand F, Nielsen K, Proux C, Rossignol T, Schein JE, Sun S, Wollschlaeger C, Wood IA, Zeng Q, Neuveglise C, Newlon CS, Perfect JR, Lodge JK, Idnurm A, Stajich JE, Kronstad JW, Sanyal K, Heitman J, Fraser JA, et al. 2014. Analysis of the genome and transcriptome of *Cryptococcus neoformans* var. *grubii* reveals complex RNA expression and microevolution leading to virulence attenuation. PLoS Genet 10:e1004261.
2. Cramer KL, Gerrald QD, Nichols CB, Price MS, Alspaugh JA. 2006. Transcription factor Nrg1 mediates capsule formation, stress response, and pathogenesis in *Cryptococcus neoformans*. Eukaryot Cell 5:1147-56.
3. Giles SS, Stajich JE, Nichols C, Gerrald QD, Alspaugh JA, Dietrich F, Perfect JR. 2006. The *Cryptococcus neoformans* catalase gene family and its role in antioxidant defense. Eukaryot Cell 5:1447-59.
4. Cox GM, Harrison TS, McDade HC, Taborda CP, Heinrich G, Casadevall A, Perfect JR. 2003. Superoxide dismutase influences the virulence of *Cryptococcus neoformans* by affecting growth within macrophages. Infect Immun 71:173-80.
5. D'Souza CA, Alspaugh JA, Yue C, Harashima T, Cox GM, Perfect JR, Heitman J. 2001. Cyclic AMP-dependent protein kinase controls virulence of the fungal pathogen *Cryptococcus neoformans*. Mol Cell Biol 21:3179-91.
6. Kidd SE, Guo H, Bartlett KH, Xu J, Kronstad JW. 2005. Comparative gene genealogies indicate that two clonal lineages of *Cryptococcus gattii* in British Columbia resemble strains from other geographical areas. Eukaryot Cell 4:1629-38.
7. Fraser JA, Giles SS, Wenink EC, Geunes-Boyer SG, Wright JR, Diezmann S, Allen A, Stajich JE, Dietrich FS, Perfect JR, Heitman J. 2005. Same-sex mating and the origin of the Vancouver Island *Cryptococcus gattii* outbreak. Nature 437:1360-4.
8. Gillum AM, Tsay EY, Kirsch DR. 1984. Isolation of the *Candida albicans* gene for orotidine-5'-phosphate decarboxylase by complementation of *S. cerevisiae ura3* and *E. coli pyrF* mutations. Mol Gen Genet 198:179-82.
